# Leukoplakia: An uncommon presentation of acute febrile neutrophilic dermatosis (Sweet syndrome)

**DOI:** 10.1016/j.jdcr.2025.08.008

**Published:** 2025-08-19

**Authors:** Suchita Sampath, Bryce DeLong, Sahil Kapur, Matthew J. Franklin

**Affiliations:** a Ohio Health Riverside Methodist Hospital, Columbus, Ohio; b The University of Toledo College of Medicine and Life Sciences, Toledo, Ohio

**Keywords:** leukoplakia, neutrophilic dermatosis, plaques, pustules, Sweet syndrome, ulcers

## Introduction

Acute febrile neutrophilic dermatosis, also known as Sweet syndrome (SS) is an uncommon inflammatory disorder characterized by dense neutrophilic infiltration, classically affecting the dermis and subcutis, with epidermal involvement only occurring secondary to pustule or ulcer formation.^1^ The dermal infiltrate is often diffuse, with papillary dermal edema and leukocytoclasis, though vasculitis is rarely present.^2^ It is typically associated with underlying conditions such as infections, hematopoietic malignancies, autoimmune disease, or inflammatory conditions, and can also be drug-induced.^1^ SS often manifests with the sudden development of painful, tender, erythematous plaques or nodules on the face, neck, and upper extremities and fever.^1^^,^^3^ Vesicles, pustules, and ulcers are sometimes observed.^1^ Oral involvement, presenting as oral ulcers, is rare in SS but occurs more frequently in patients with hematologic disorders.^1^ A skin biopsy demonstrating cutaneous neutrophilic infiltration is essential for making the diagnosis.^3^ We present a rare case of acute febrile neutrophilic dermatosis with oral mucosa involvement that developed following a presumed viral infection.

## Case presentation

**Fig 1:**
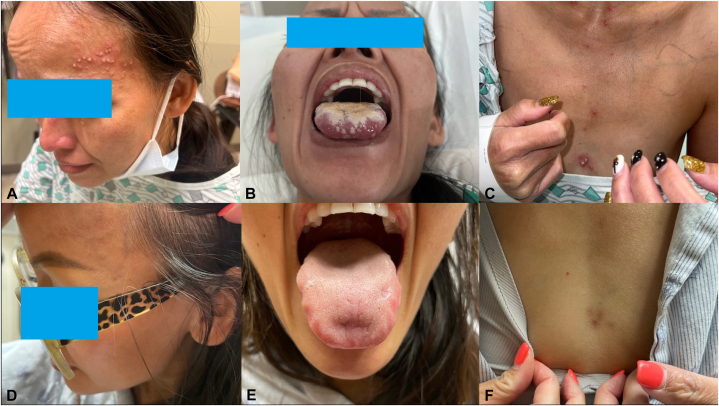
**A,** Pustules on the left temporal region; **(B)** Oral pustules, leukoplakia, and ulcers on the dorsal tongue; **(C)** Scattered pustules along the mediastinum. **D** and **E,** Patient after 4 weeks of 40 mg prednisone, showing complete resolution of temporal pustules **(D)**, oral ulcers and leukoplakia **(E)**, and chest pustules **(F)**.

A 41-year-old female presented with oral ulcers and pustules on her left temple, neck, chest, and back (Fig 1, *A-C*). Her symptoms began 20 days earlier with myalgias, shortness of breath, bitemporal headache, and fever after returning from the Dominican Republic. Urgent care testing was negative for COVID-19 and influenza. Doxycycline was prescribed for presumed pneumonia. Two days later, she presented with worsening throat pain, dysphagia, and new tongue leukoplakia with underlying ulcerations. The patient was discharged with nystatin for oral candidiasis. The same day, the patient presented to another emergency department with severe pain. The patient underwent an infectious workup, which was negative for syphilis, HIV, and oral candidiasis, and was admitted for otolaryngology evaluation. The patient received broad-spectrum antibiotics, steroids, and intravenous hydration. Valacyclovir was initiated for treatment of presumed herpes simplex virus infection. The patient noted some improvement with oral prednisone and dexamethasone oral rinse. Additional evaluations, including HSV polymerase chain reaction swab, cytomegalovirus serology, and an acute hepatitis panel, were negative. The patient was discharged with symptomatic care.

**Fig 2:**
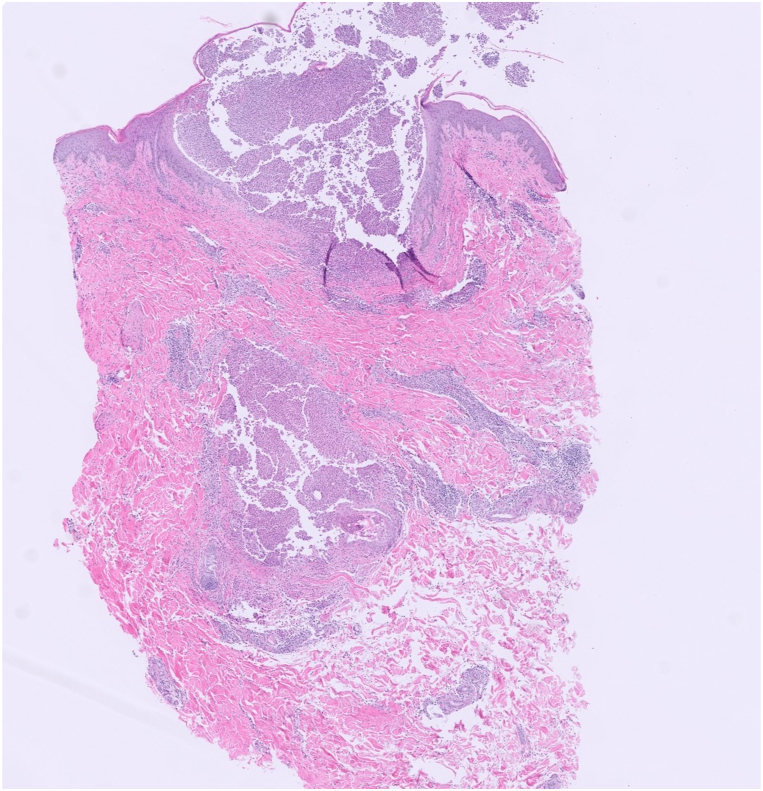
H&E 20×. A punch biopsy of a pustule on the right breast demonstrates disruption of the epidermis with intraepidermal and intradermal aggregates of inflammatory cells. Vasculitis is not identified.

**Fig 3:**
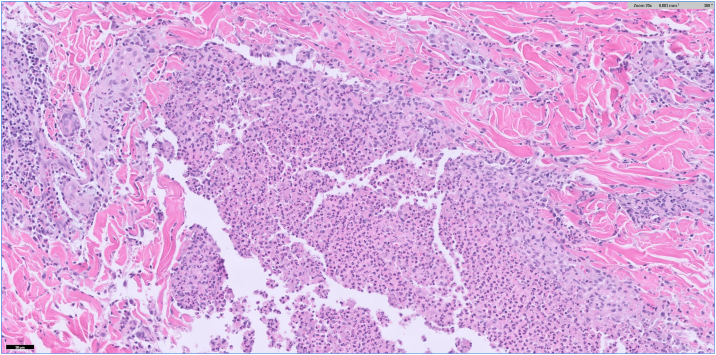
H&E 200×. Examination of the dermal infiltrate reveals innumerable neutrophils with admixed lymphocytes and rare eosinophils.

**Fig 4:**
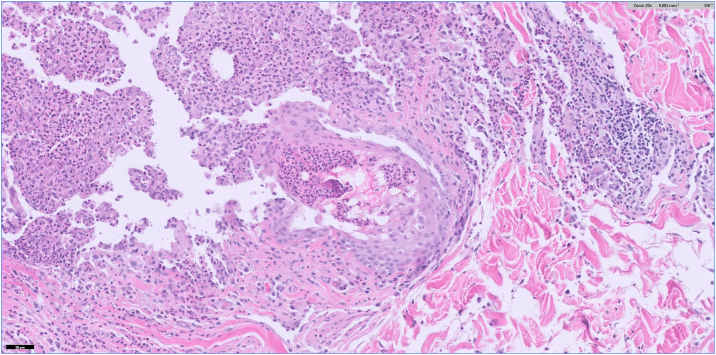
H&E 200×. Dermal neutrophilic abscess with disruption of a follicle. Scattered leukocytoclasis is present.

After discharge, the patient developed scattered, painful, pruritic pustules involving the left temple, neck, chest, and back, in addition to the leukoplakia. Of note, there was no genital involvement. This prompted a return to the hospital, and she was admitted for infectious disease and dermatology evaluation. Bloodwork on this admission demonstrated an elevated C-reactive protein (23.2 mg/L) and erythrocyte sedimentation rate (44 mm/hr); A complete blood count demonstrated leukocytosis (12.09 K/mcL) with elevated absolute neutrophil count (9.75 K/mcL). Repeat wound cultures from the dorsal tongue and from a ruptured cutaneous pustule were collected, as well as a punch biopsy of an intact pustule from the left breast. The wound cultures showed normal skin and oral flora without pathogenic yeast. Histologic examination of the punch biopsy demonstrated a dense dermal neutrophilic infiltrate with intraepidermal pustule formation (Fig 2, Fig 3, Fig 4). Periodic acid-Schiff and Gram stains were negative for fungi and bacteria, respectively. Focal karyorrhexis and leukocytoclasis were present, though frank vasculitis was not identified. The patient was diagnosed with a neutrophilic dermatosis with atypical features and started on high-dose corticosteroids. Our differential diagnoses included atypical acute febrile neutrophilic dermatosis (SS), pyoderma gangrenosum with pustular morphology, Behçet's disease, neutrophilic mucositis, and subcorneal pustular dermatosis. Despite atypical findings, we regarded a provisional diagnosis of SS to be most appropriate. The patient was screened for an occult underlying hematologic malignancy with a peripheral blood smear, which demonstrated a normal white blood cell count without evidence of blasts or atypical lymphocytes. It was suspected that this eruption was secondary to an upper respiratory infection acquired during vacation. The patient was discharged on high-dose steroids with outpatient dermatology follow-up. At the 4-week follow-up appointment, the patient had complete resolution of oral and skin lesions as well as the associated odonyphagia (Fig 1, *D-F*). She was instructed to complete the steroid taper without the implementation of a steroid-sparing agent.

## Discussion

SS, also known as acute febrile neutrophilic dermatosis, was first described in 1964 by Robert Douglas Sweet.^4^ Since then, SS has been classified into 3 major subtypes: classical or idiopathic, malignancy-associated, and drug-induced.^3^

This disease classically presents with an acute onset of fever, leukocytosis, and painful, erythematous, edematous papules and plaques.^4^ Patients may also experience fatigue, headache, malaise, and myalgias.^1^ Involvement of extracutaneous sites, including the lungs and eyes, has been described. In rare cases, patients may present with ulcerative oral lesions, which may be associated with hematologic disorders and malignancies.^1^^,^^5^

SS typically presents in the third through sixth decade of life, with peak incidence in the Spring and Fall.^1^^,^^6^ The female-to-male ratio is approximately 4:1.^1^^,^^6^

The exact pathogenesis remains unclear. One hypothesis proposes that SS is a hypersensitivity reaction elicited by the exposure of a novel antigen in the setting of underlying disease, such as malignancy.^4^^,^^7^ In this model, there is dysregulation of inflammatory cytokines, interleukins, granulocyte colony-stimulating factor, and granulocyte-macrophage colony-stimulating factor.^3^^,^^4^^,^^7^ Supporting this hypothesis are studies demonstrating increased expression of inflammatory markers such as myeloperoxidase and metalloproteinases 7, as well as the occurrence of SS following administration of granulocyte colony-stimulating factor and granulocyte-macrophage colony-stimulating factor 2,3,6.

Su and Liu's initial diagnostic criteria, which included 2 major and 4 minor criteria.^8^ While their 2 major criteria are still widely accepted, the minor criteria were modified by von den Driesch.^8^ In classic SS, both major criteria and 2 of the 4 minor criteria are needed to establish the diagnosis (Table I).^8^ It is important to note that several clinical (eg generalized pustular, subcutaneous) and histologic (cryptococcoid, lymphocytic, histiocytoid) variants of SS are recognized, which may create diagnostic difficulty when conventional features of SS are absent.

**Table I:** Proposed diagnostic criteria of Sweet syndrome: both major criteria required and 2 of the 4 minor criteria need to be met for the diagnosis^1^

Major criteria	Minor criteria
1. Abrupt tender lesions	1. Fever >38 °C
2. Neutrophilic infiltrate on biopsy (no vasculitis)	2. Association with malignancy/infection/drug
	3. Lab: ESR >20 mm/h, CRP+, leukocytosis >8k, neutrophils >70%
	4. Excellent response to treatment with systemic corticosteroids or potassium iodide

*CRP*, C-reactive protein; *ESR*, erythrocyte sedimentation rate.

Though treatment of the underlying trigger of SS, if identified, may result in disease regression, the gold standard of treatment remains systemic corticosteroids.^9^ In drug-induced SS, cessation of the offending medication typically results in disease resolution once the drug has been cleared.^10^ In localized disease, intralesional corticosteroid injections and topical corticosteroids may be used.^1^ For those patients unable to tolerate corticosteroids or who fail to respond, alternative treatment recommendations include potassium iodide and colchicine.^9^ Second-line agents, including dapsone, indomethacin, clofazimine, and cyclosporin, can be used in refractory disease for those who do not respond to preferred agents.^9^^,^^10^

Overall, acute neutrophilic dermatosis has a good prognosis. With appropriate treatment, the eruption resolves without scarring.^1^ Early disease management and treatment can also prevent complications such as secondary infection.^10^ Recurrence rates as high as 50% in patients with underlying hematologic malignancy or inflammatory disease have been reported.^1^ Awareness of a potential underlying malignancy or myelodysplastic syndrome is crucial in patients with unusual clinical findings in SS, such as oral ulcers.^1^ Screening with a CBC and peripheral blood smear should be performed and followed up with a bone marrow biopsy if the smear is abnormal to rule out AML and other myeloproliferative disorders.

## Conflicts of interest

None disclosed.

## References

[1] Vashisht P., Goyal A., Hearth Holmes M.P. (2025). StatPearls.

[2] Kazlouskaya V., Junkins-Hopkins J.M. (2018). Lymphocytes in Sweet syndrome: a potential diagnostic pitfall. J Cutan Pathol.

[3] Villarreal-Villarreal C.D., Ocampo-Candiani J., Villarreal-Martínez A. (2016). Sweet syndrome: a review and update. Actas Dermosifiliogr.

[4] Raza S., Kirkland R.S., Patel A.A., Shortridge J.R., Freter C. (2013). Insight into Sweet's syndrome and associated-malignancy: a review of the current literature. Int J Oncol.

[5] Cohen P.R., Kurzrock R. (2000). Sweet's syndrome: a neutrophilic dermatosis classically associated with acute onset and fever. Clin Dermatol.

[6] Chaudhry A.R., Iftikhar I., Choudhry S.A., Islam R., Islam H. (2023). Fluoroquinolone-induced Sweet syndrome: a case report. Cureus.

[7] Paydas S. (2013). Sweet's syndrome: a revisit for hematologists and oncologists. Crit Rev Oncol Hematol.

[8] von den Driesch P. (1994). Sweet's syndrome (acute febrile neutrophilic dermatosis). J Am Acad Dermatol.

[9] Khan U., Rizvi H., Ali F., Lebovic D. (2016). Sweet syndrome: a painful reality. BMJ Case Rep.

[10] Cohen P.R. (2007). Sweet's syndrome–a comprehensive review of an acute febrile neutrophilic dermatosis. Orphanet J Rare Dis.

